# Brain Injury Awareness Month — March 2019

**DOI:** 10.15585/mmwr.mm6810a1

**Published:** 2019-03-15

Brain Injury Awareness Month, observed each March, was established 3 decades ago to educate the public about the incidence of brain injury and the needs of persons with brain injuries and their families (*1*). Caused by a bump, blow, or jolt to the head, or penetrating head injury, a traumatic brain injury (TBI) can lead to short- or long-term changes affecting thinking, sensation, language, or emotion.

A report in this issue of *MMWR *found that during 2010–2016, nearly 2 million children had a TBI-related emergency department visit because of sports- and recreation-related activities (*2*). TBIs associated with football, bicycling, playground activities, basketball, and soccer contributed to the majority of these visits (*2*).

Brain Injury Awareness Month is an opportunity to encourage broader implementation of evidence-based practices to reduce pediatric TBIs and their sequelae. Primary prevention efforts aimed at the leading causes of TBI among children are critical. If a TBI occurs, CDC supports the development of return to activity plans by health care providers, customized to a child’s symptoms, as well as linkages to services for children with persistent symptoms to promote positive health outcomes (*3*,*4*). Additional information is available at https://www.cdc.gov/traumaticbraininjury.
